# Selective inhibition of GluN2D-containing N-methyl-D-aspartate receptors prevents tissue plasminogen activator-promoted neurotoxicity both in vitro and in vivo

**DOI:** 10.1186/1750-1326-6-68

**Published:** 2011-10-05

**Authors:** Amandine Jullienne, Axel Montagne, Cyrille Orset, Flavie Lesept, David E Jane, Daniel T Monaghan, Eric Maubert, Denis Vivien, Carine Ali

**Affiliations:** 1 INSERM U919, Serine Proteases and Pathophysiology of the neurovascular Unit, Cyceron, University of Caen Basse-Normandie, Caen, France; 2 Department of Physiology and Pharmacology, Medical Research Center for Synaptic Plasticity, University of Bristol, Bristol, UK; 3 Department of Pharmacology and Experimental Neuroscience, University of Nebraska Medical Center, Omaha, Nebraska 68198-5800, USA

**Keywords:** Tissue plasminogen activator, GluN2D, UBP145, excitotoxicity, stroke, glutamatergic neurotransmission

## Abstract

**Background:**

Tissue plasminogen activator (tPA) exerts multiple functions in the central nervous system, depending on the partner with which it interacts. In particular, tPA acts as a positive neuromodulator of N-methyl-D-aspartate glutamatergic receptors (NMDAR). At the molecular level, it has been proposed that the pro-neurotoxicity mediated by tPA might occur through extrasynaptic NMDAR containing the GluN2D subunit. Thus, selective antagonists targeting tPA/GluN2D-containing NMDAR signaling would be of interest to prevent noxious effects of tPA.

**Results:**

Here, we compared three putative antagonists of GluN2D-containing NMDAR and we showed that the new compound UBP145 ((2R*,3S*)-1-(9-bromophenan-threne-3-carbonyl)piperazine-2,3-dicarboxylic acid) is far more selective for GluN2D subunits than memantine and PPDA (phenanthrene derivative (2S*, 3R*)-1-(phenanthrene-2-carbonyl)piperazine-2,3-dicarboxylic acid). Indeed, in vitro, in contrast to the two other compounds, UBP145 prevented NMDA toxicity only in neurons expressing GluN2D (ie, in cortical but not hippocampal neurons). Furthermore, in cultured cortical neurons, UBP145 fully prevented the pro-excitotoxic effect of tPA. In vivo, we showed that UBP145 potently prevented the noxious action of exogenous tPA on excitotoxic damages. Moreover, in a thrombotic stroke model in mice, administration of UBP145 prevented the deleterious effect of late thrombolysis by tPA.

**Conclusions:**

In conclusion, tPA exerts noxious effects on neurons by acting on GluN2D-containing NMDAR and pharmacological antagonists of GluN2D-containing NMDAR could be used to prevent the ability of tPA to promote neurotoxicity.

## Background

Tissue plasminogen activator (tPA) is a serine protease initially described to promote vascular fibrinolysis by converting plasminogen into active plasmin [1]. But tPA is not just a vascular protease, since it is also expressed by nerve cells and exerts important functions in the brain parenchyma [2,3]. For instance, during perinatal development, tPA influences neuronal migration and synaptic outgrowth [4,5], while in the adult brain, tPA controls synaptic plasticity [6], long-term potentiation [7] and long-term depression [8]. In preclinical models of brain disorders, it has been shown that tPA can differentially influence the fate of nerve cells, due to its pleiotropic actions and targets that include, excitotoxicity, apoptosis, neuroinflammation or myelination [9-13].

The pleiotropic functions of tPA in the central nervous system can be explained by several mechanisms. Among them, there is strong evidence that the direct or indirect interaction of tPA with the N-methyl-D-aspartate receptor (NMDAR) exerts a positive neuromodulatory action [10,14,15]. tPA can thus increase NMDA-evoked calcium entry, which can be trophic under physiological conditions, or alternatively noxious under excitotoxic conditions. NMDARs are ion-gated glutamate receptors, mainly described as heterotetrameric channels composed of two GluN1 subunits and two GluN2 subunits (in some cases, NMDARs may also contain GluN3 subunits [16,17]). GluN1 and GluN2 subunits exist as isoforms, explaining the varied electrophysiological properties of NMDARs [18]. NMDARs differentially control neuronal outcome (trophism versus neurotoxicity) depending on their neuronal localization (synaptic and extrasynaptic) [19,20], their subunit composition [21] and/or their anchorage with cytoplasmic or membrane-associated proteins [22].

We have recently demonstrated that the pro-excitotoxic effects of tPA could be blocked by the co-application of the weakly-selective GluN2D antagonist, PPDA (phenanthrene derivative (2S*, 3R*)-1-(phenanthrene-2-carbonyl)piperazine-2,3-dicarboxylic acid) or silencing of GluN2D [23]. PPDA is 2- to 5-fold more selective for GluN2C- or GluN2D-containing NMDARs than for receptors containing either GluN2A- or GluN2B subunits [24,25]. By extending the structure-activity analysis around the PPDA structure, a brominated homolog (UBP145; (2R*,3S*)-1-(9-bromophenanthrene-3-carbonyl)piperazine-2,3-dicarboxylic acid) has been recently produced and characterized as being more selective than PPDA, as UBP145 displays a 7- to 17- fold selectivity for GluN2D-containing NMDARs over GluN2B- or GluN2A-containing NMDARs [26]. Interestingly, it has also been suggested that memantine, used as Alzheimer's disease medication, may act as an antagonist of GluN2D-containing NMDAR [27].

In the present study, we investigated in vitro and in vivo, whether putative antagonists of GluN2D-containing NMDAR (memantine, PPDA and UBP145) could prevent the aggravating effect of tPA on NMDAR-mediated neurotoxicity. In vitro, we show that UBP145 is more selective than memantine or PPDA, as during excitotoxic challenges, it acted only on neurons expressing GluN2D. Accordingly, in vivo, UBP145 prevents the deleterious effects of exogenous tPA against excitotoxicity and ischemic stroke.

## Results

### UBP145 selectively prevents tPA-promoted neurotoxicity mediated by GluN2D-containing NMDA receptor

**Figure 1:**
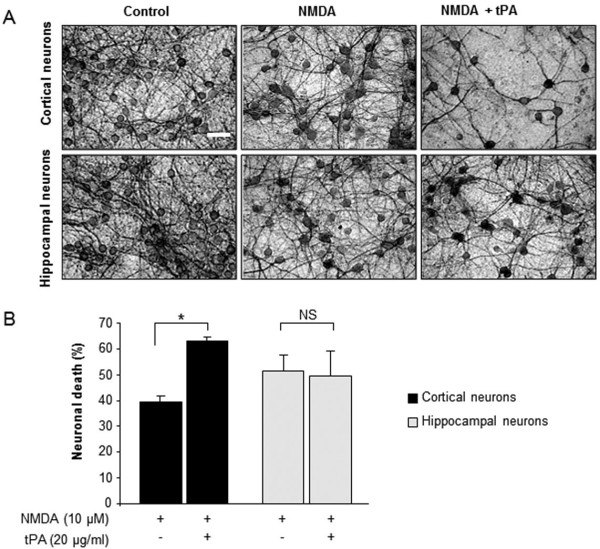
**tPA increases NMDA-mediated neurotoxicity in cortical neuronal cultures but not in hippocampal neuronal cultures**. Cortical and hippocampal neurons were exposed to NMDA (10 μM) with or without tPA (20 μg/mL) for 24 hours. (A) Bright field photomicrographs of cultures stained by peroxidase with a MAP2 immunostaining, scale bar 100 μm. (B) Quantification of neuronal death by LDH release: tPA increased excitotoxic neuronal death in cortical but not in hippocampal cultures (n = 12; *p < 0.05; NS: not significant).

In a paradigm of rapidly triggered excitotoxicity (exposure to a high dose of NMDA for 1 hour and measurement of neuronal death 24 hours later), we have previously shown that tPA potentiates neuronal death of cortical neurons but not of hippocampal neurons. This differential sensitivity of cortical versus hippocampal neurons was respectively attributed to the presence and absence of GluN2D subunits in NMDAR at both the mRNA and protein levels [23]. Here, we first extended our previous observations by investigating the sensitivity to tPA of cortical and hippocampal neurons in vitro, in a paradigm of slowly triggered excitotoxicity (exposure to a low dose of NMDA for 24 h). As reported for rapidly triggered excitotoxicity [23], slowly triggered excitotoxic cell death was equivalent in both types of neurons, but tPA (20 μg/ml) increased excitotoxic neuronal death in cortical cultures but not in hippocampal cultures (see MAP2 immunostaining in Figure 1A and the corresponding quantification of neuronal death in Figure 1B; +30%, n = 12, p < 0.05). Our next step was to compare three pharmacological agents reported to be more or less selective antagonists of GluN2D, for their ability to prevent tPA-mediated pro-neurotoxicity: memantine [27,28], PPDA (phenanthrene derivative (2S*, 3R*)-1-(phenanthrene-2-carbonyl)piperazine-2,3-dicarboxylic acid) [24]and UBP145 ((2R*,3S*)-1-(9-bromophenan-threne-3-carbonyl)piperazine-2,3-dicarboxylic acid) [26]. We took advantage of the lack of expression of the GluN2D in cultured hippocampal neurons when compared to cortical neurons and we postulated that a molecule preferentially targeting the pro-neurotoxicity effects of tPA: i/should display a dose-dependent inhibitory effect on NMDA-mediated neurotoxicity in cultured cortical neurons, ii/should not influence NMDA-mediated neurotoxicity in cultured hippocampal neurons; iii/should prevent the aggravating effect of tPA on NMDA-mediated neurotoxicity in cultured cortical neurons, at a concentration not influencing NMDA alone-mediated neurotoxicity. The doses of memantine, PPDA and UBP145 were chosen according to their published affinity values [24,26,27].

**Figure 2:**
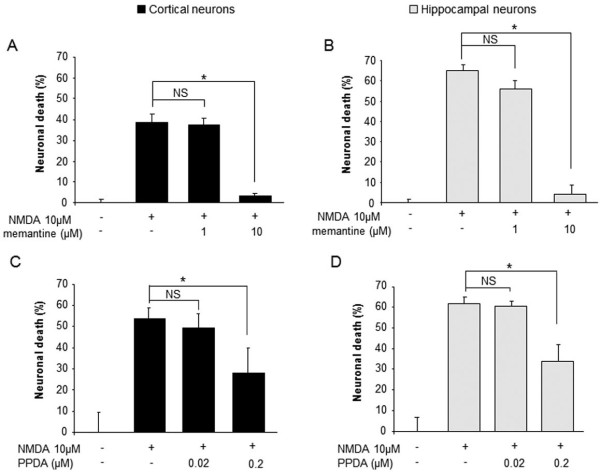
**Memantine and PPDA protect neurons from NMDA-mediated neurotoxicity in both cortical and hippocampal cultures**. Cortical (A, C) and hippocampal (B, D) neurons were exposed to NMDA (10 μM) with or without memantine (A, B) or PPDA (C, D). At the lowest doses, memantine (1 μM) and PPDA (0.02 μM) failed to protect both types of neurons, but at the highest doses, memantine (10 μM) and PPDA (0.2 μM) were able to protect cortical and hippocampal neurons from excitotoxicity (A: n = 12, B: n = 11, C: n = 16, D: n = 9; *p < 0.05; NS: not significant).

Memantine was tested for its ability to influence NMDA-mediated neurotoxicity in both cultured cortical (Figure 2A) and hippocampal neurons (Figure 2B) at 1 and 10 μM. The lowest dose of memantine (1 μM) had no effect on the extent of NMDA-induced neuronal loss in cortical (n = 12, p < 0.05, Figure 2A) and hippocampal (n = 12, p < 0.05, Figure 2B) neurons. By contrast, at the highest dose (10 μM), memantine nearly fully prevented NMDA-mediated neuronal death in both cortical (n = 12, p < 0.05, Figure 2A) and hippocampal (n = 12, p < 0.05, Figure 2B) neurons. Since memantine displayed the same profile of inhibition of NMDA-mediated neuronal death in cortical and hippocampal neurons, memantine did not meet our second selection criterion.

PPDA (from 0.02 to 2 μM) was tested in the same paradigm and like memantine, PPDA showed a comparable dose-dependent profile of inhibition of NMDA-mediated neurotoxicity in both cultured cortical (Figure 2C) and hippocampal neurons (Figure 2D). Thus, the pharmacological profile of PPDA (similar potency to prevent NMDA-mediated excitotoxicity in cortical and hippocampal cultures) did not fit with our criteria.

**Figure 3:**
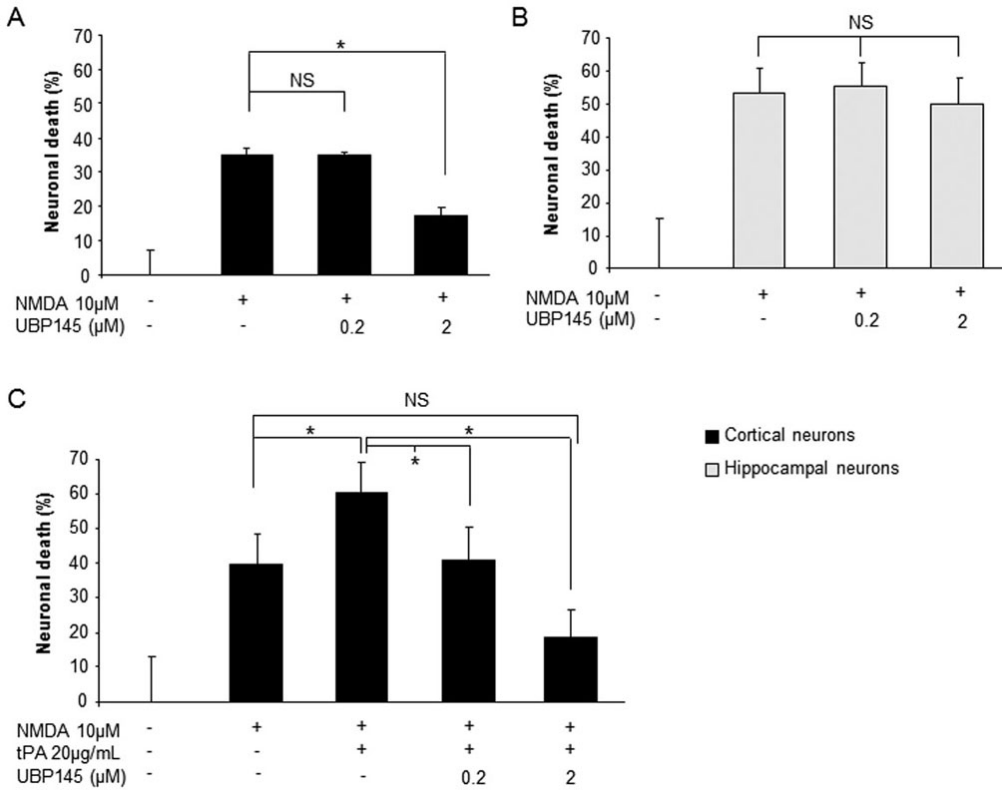
**UBP145 has no effect on NMDA-mediated neurotoxicity in hippocampal neurons but prevents tPA-induced potentiation of NMDA-mediated neurotoxicity in cortical neurons**. Cortical (A, C) and hippocampal (B) neurons were exposed to NMDA (10 μM) with or without UBP145 and tPA (20 μg/mL) for 24 hours. Quantification of neuronal death by LDH release in the medium: (A, B) UBP145 2 μM protected cortical but not hippocampal neurons from neurotoxicity, (C) although UBP145 0.2 μM did not protect cortical neurons from excitotoxicity, it was able to prevent tPA-induced potentiation of excitotoxicity (n = 12; *p < 0.05; NS: not significant).

**Figure 4:**
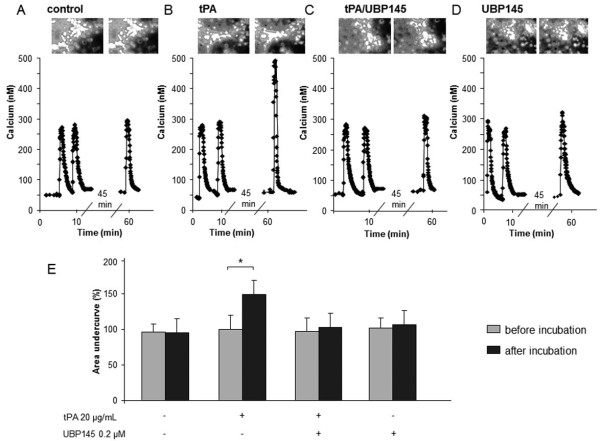
**UBP145 prevents tPA-promoted NMDA-induced Ca**^**2+ **^**influx in cortical neurons**. Two successive 30 second exposures to NMDA (50 μM) lead to a rapid and reproducible Ca^2+ ^influx in cultured cortical neurons (A-D; n = 3 independent cultures; n = 150 cells). Cells were then incubated for 45 minutes with tPA alone (20 μg/mL) or with UBP145 0.2 μM. NMDA exposure was performed again. In contrast to tPA alone (B, E), when co-incubated with UBP145, tPA did not promote NMDA-induced Ca^2+ ^influx in cortical neurons (C, E). UBP145 alone at 0.2 μM did not affect NMDA-induced Ca^2+ ^influx (D, E). For each condition, illustrative images (during NMDA stimulation before and after treatment) are provided at the top of calcium recordings (A-D).

Finally, we tested the more selective GluN2D antagonist, UBP145 [26] at 0.2 and 2 μM. Interestingly, although UBP145 at 2 μM partially reduced NMDA-mediated neurotoxicity in cultured cortical neurons (-50%, n = 12, p < 0.05, Figure 3A), it had no effect in cultured hippocampal neurons (Figure 3B). Moreover, at 0.2 μM, a dose without effect on NMDA-mediated neurotoxicity (Figure 3C), UBP145 completely prevented tPA-induced potentiation of NMDA-mediated neurotoxicity in cultured cortical neurons (n = 12, p < 0.05, Figure 3C). Consistent with the observation that UBP145 prevents tPA-induced aggravation of NMDA toxicity, calcium video-imaging of cortical neurons indicated that the co-incubation of UBP145 (0.2 μM) completely abolished the promoting action of tPA (20 μg/ml) on NMDA-evoked calcium influx (Figure 4B versus 4C, Figure 4E). By itself, UBP145 did not modify NMDA-evoked calcium influx (Figure 4D). Thus, UBP145 displays all the criteria of a good candidate to prevent the pro-neurotoxicity of tPA that occur through the GluN2D subunit of NMDAR.

### In an in vivo excitotoxic challenge, the inhibition of GluN2D-containing NMDAR can fully prevent the pro-excitotoxic effect of intravenously administered tPA

**Figure 5:**
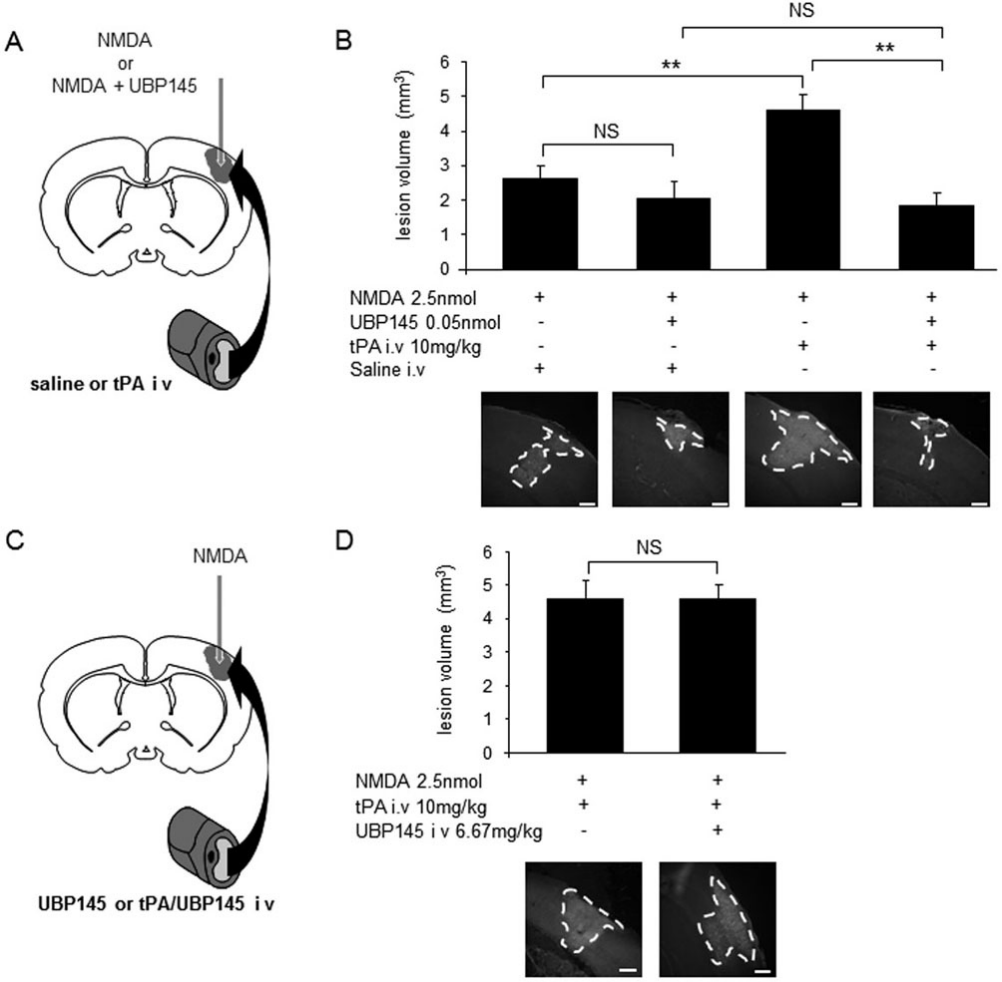
**In vivo, UBP145 can fully prevent the pro-excitotoxic effect of intravenously administered tPA**. Cortical injections of NMDA (2.5 nmoles; in 0.33 μl), alone or with UBP145 (0.05 nmoles) were performed in mice using a stereotaxic frame. (A) When UBP145 was co-injected with NMDA, it prevented tPA-induced potentiation of the excitotoxic lesion (n = 6 per group, **p < 0.01; NS: not significant). (B) When UBP145 was administered intravenously, it was not able to reduce NMDA-induced excitotoxic lesion (n = 5 per group; NS: not significant). Scale bar: 400 μm

Based on our in vitro data, the ability of UBP145 to prevent the pro-neurotoxicity of tPA was tested in mice, in a model of NMDA-induced excitotoxicity in the cortex. The dose of tPA was chosen according to our previous works [10]. For its intravenous administration, the dose of UBP145 was chosen to respect the molar ratio UBP145/tPA shown to be efficient in our in vitro studies (Figure 4), while for its intra-structure administration, the dose of UBP145 was similar to that of PAI-1 (an inhibitor of tPA) known to be effective in preventing the noxious effect of intravenous tPA in the in vivo excitotoxic model [29]. NMDA alone induced a lesion restricted to the cortical area (n = 6). When injected intravenously, tPA (10 mg/kg) doubled the NMDA-induced cortical lesion (n = 6, p < 0.01) (Figure 5A,B). In agreement with our in vitro observations, the cortical co-injection of UBP145 completely prevented the noxious effect of tPA administration on NMDA-mediated excitotoxicity (n = 6, p < 0.01) (Figure 5A). In contrast to the neuroprotective effect of UBP145 when administered inside the lesion site, the intravenous administration of UBP145 (6.67 mg/kg) failed to prevent the pro-excitotoxic effect of exogenous tPA in our paradigm of cortical excitotoxicity (Figure 5C,D). Our observations suggest that despite being a potent and selective blocker of the deleterious effect of tPA on GluN2D containing NMDAR, UBP145 does not cross the intact blood-brain barrier (BBB).

### In an in vivo model of thrombotic stroke, the deleterious effect of late thrombolysis by tPA is reversed by UBP145, an antagonist of the GluN2D-containing NMDAR

**Figure 6:**
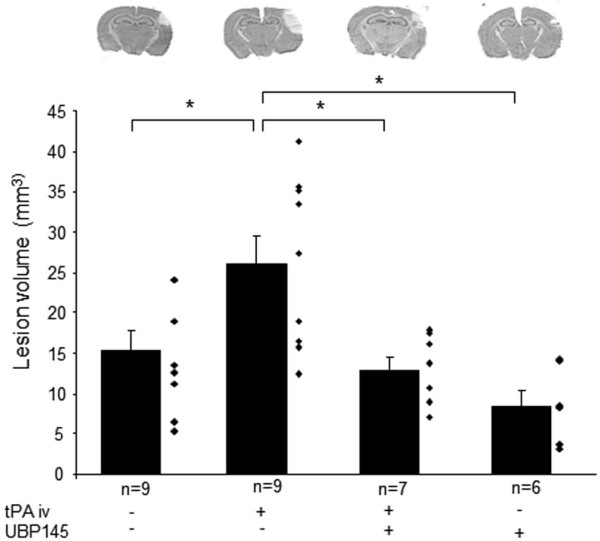
**UBP145 reverses the deleterious effect of late thrombolysis by tPA in an in vivo model of thrombotic stroke**. Thrombotic stroke was induced in mice by injection of thrombin (1 UI.) into the middle cerebral artery. Late intravenous thrombolysis by tPA (10 mg/kg) alone or in combination with UBP145 (6.67 mg/kg) was performed 4 hours after MCA occlusion. The co-administration of UBP145 with tPA prevented the deleterious effects of late thrombolysis with tPA (*p < 0.05). Representative thionine-stained sections are shown for each condition.

We have developed and fully characterized a model of thrombotic stroke in mice by injection of thrombin into the middle cerebral artery, leading to the formation of a local thrombus [30-32]. In this model, as in the clinical setting, early thrombolysis by intravenous injection of tPA is beneficial, while late thrombolysis by tPA is deleterious and increases BBB leakage [32]. We thus tested the effect of intravenous UBP145 (6.67 mg/kg) in this model of thrombotic stroke coupled or not to a late thrombolysis by tPA (10 mg/kg). In these experimental conditions, we have previously demonstrated an opening of the BBB [32]. Accordingly, the lesion volumes in mice receiving late thrombolysis by tPA were higher than the ones measured in mice with no tPA treatment (Figure 6, +70%, n = 9, p < 0.05). When injected intravenously 4 hours after clot formation, although UBP145 did not influence the ischemic lesion in the absence of tPA, the deleterious effect of late thrombolysis (tPA iv, 4 hours after clot formation) was completely prevented by the co-injection of UBP145 (n = 7, p < 0.05).

## Discussion

We have previously demonstrated that tPA is pro-excitotoxic in cortical neurons but not in hippocampal neurons. We also evidenced by using both pharmacological and molecular approaches that this discrepancy could be due to a selective action of tPA on extra-synaptic NMDAR containing the GluN2D subunit [23]. These observations support the ideas that there are several subpopulations of NMDAR and that these subpopulations exert differential and even sometimes opposite actions (such as trophic versus neurotoxic effects. These differential actions of NMDAR can be at least in part attributed to variations in their subunit composition and/or recruitment of different intracellular signalling pathways. To date, this idea had been mainly investigated for GluN2A and GluN2B [21,33,34], but little is known regarding other GluN2 variants. Thus, further investigating the relative contribution of different NMDAR populations is relevant for many physiological/pathological situations, including the ones involving tPA and/or GluN2D, such as learning/memory processes [35-37], food intake [38], stroke [3,23], Alzheimer's disease [39,40] or nociception [41]. We thus sought here to characterize among the currently available drugs suggested to preferentially act on GluN2D-containing NMDAR (ie, memantine, PPDA and UBP145), which one is the more efficient in targeting the potentiating effects of tPA on NMDAR-mediated neuronal death.

In the United States and Europe, memantine is indicated to treat patients with moderate to severe Alzheimer's disease. Recently, it has been reported that in the presence of 1 mM magnesium, memantine becomes 6-8 fold more selective for GluN2C- or GluN2D-containing receptors over those with either GluN2A or GluN2B subunits [28]. However, in our hands, memantine was as efficient in preventing NMDA neurotoxicity in neurons expressing (cortical) or not expressing (hippocampal) GluN2D.

We then used another weakly-selective GluN2D competitive antagonist, PPDA [24,25] and the more selective agent, UBP145 [26]. Much lower doses of PPDA than those of memantine were used, but like memantine, PPDA displayed a similar potency to prevent NMDA-mediated excitotoxicity in cortical and hippocampal cultures. By contrast, we found that low doses of UBP145 could differentially affect NMDA-induced death of cortical and hippocampal neurons that respectively express and do not express GluN2D. UBP145 thus appears as a sufficiently-selective pharmacological antagonist of GluN2D as to selectively protect GluN2D-expressing cortical neurons. At a very low dose -not influencing the extent of NMDA-induced excitotoxicity-, UBP145 prevented tPA from exacerbating neuronal death. Consistent with this, tPA was unable to increase NMDA-evoked calcium influx when co-administered with UBP145. These in vitro data argue again that GluN2D-containing NMDAR are the critical effectors of the pro-excitotoxic effects of tPA. From a molecular point of view, this is in agreement with the demonstrations that neurotoxicity is mediated by extra-synaptic NMDAR [19] and that GluN2D is exclusively distributed in extra-synaptic NMDAR [37].

We then used UBP145 in vivo in an attempt to prevent the deleterious action of tPA during excitotoxic challenges. First, we observed that UBP145 can block the pro-excitotoxic effect of exogenous tPA (administered intravenously) in a model of cortical excitotoxicity. However, this prevention requires that UBP145 is administered in the brain parenchyma, as no effect was observed for an intravenous injection. This suggests that in this model that does not induce blood-brain barrier leakage [29], UBP145 cannot extravasate into the brain parenchyma. Yet, in a model of thrombotic stroke highly relevant to the clinical setting [30-32], we found that UBP145 co-injected intravenously with tPA fully prevented the noxious action of late tPA-mediated thrombolysis on the extent of ischemic brain damage. This strongly suggests that, in conditions of ischemic brain injury, in which the integrity of the BBB is altered [30-32], the GluN2D-containing NMDAR antagonist UBP145, could be used as an adjunct therapy to increase the therapeutic window of tPA-mediated thrombolysis that today, is restricted to the first 4.5h after stroke onset [42].

## Conclusion

In conclusion, we provide here strong evidence that tPA influences neurotoxicity selectively through GluN2D-containing NMDAR and we provide support for the use of antagonists like UBP145 in order to selectively prevent the deleterious effects of tPA on NMDAR-mediated pathological functions.

## Methods

### Materials

NMDA, memantine and PPDA were purchased from Tocris (Bristol, UK). Human recombinant tPA (rtPA; Actilyse^®^) was purchased from Boehringer Ingelheim (Paris, France). Dulbecco's modified Eagle's medium (DMEM), poly-D-lysine, laminin, glutamine, cytosine β-D-arabinoside, HEPES-buffered saline solution (HBBSS) and glycine were purchased from Sigma-Aldrich (L'Isle d'Abeau, France). UBP145 was produced as previously described [12].

### Neuronal cultures

Neuronal cultures were prepared from Swiss mouse embryos (embryonic day 15-16) as described earlier [23]. Cortices or hippocampi were dissected and dissociated in DMEM, and plated, respectively, on 24- and 48-well plates previously coated with poly-D-lysine (0.1 mg/ml) and laminin (0.02 mg/ml). Cells were cultured in DMEM supplemented with 5% foetal bovine serum, 5% horse serum (both from Invitrogen, Cergy Pontoise, France) and 2 mM glutamine. Cultures were maintained at 37°C in a humidified 5% CO_2 _atmosphere. To inhibit glial proliferation, cytosine ß-D-arabinoside (10 μM) was added after 3 or 1 day(s) in vitro (DIV) in cortical and hippocampal cultures, respectively.

### Excitotoxic neuronal death

Excitotoxicity was induced at 12-13 DIV by exposure to NMDA (10 μM) in serum-free DMEM supplemented with 10 μM of glycine for 24 hours. NMDA was applied alone or together with rtPA (20 μg/ml) and/or antagonists. After 24 hours, neuronal death was estimated by Microtubule-associated protein 2 (MAP2) immunostaining and quantified by measurement of the activity of lactate dehydrogenase (LDH) released from damaged cells into the bathing medium with a cytotoxicity detection kit (Roche Diagnostics, Mannheim, Germany). The LDH level corresponding to the maximal neuronal death was determined in sister cultures exposed to 200 μM NMDA. Background LDH levels were determined in sister cultures subjected to control washes. Experimental values were measured after subtracting LDHmin and then normalized to LDHmax-LDHmin to express the results in percentage of neuronal death.

### Calcium videomicroscopy

Experiments were performed at room temperature on the stage of a Nikon Eclipse inverted microscope equipped with a 75 W Xenon lamp and a Nikon 40x, 1.3 numerical aperture epifluorescence oil immersion objective. Cell cultures were transferred into a serum free medium (HBBSS) and loaded with fura-2/AM 10 μM (Invitrogen) for 45 minutes at 37°C. Neurons were washed and NMDA treatment (1 mL at 50 μM) was applied using a peristaltic pump (2 mL/minute). UBP145 (0.2 μM) and/or tPA (20 μg/mL) were directly applied in the medium and incubated for 45 minutes. Fura-2 (excitation: 340, 380 nm, emission: 510 nm) ratio images were acquired with a CCD camera (Princeton Instrument, Trenton, New Jersey), and digitized (256 × 512 pixels) using Metafluor 4.11 software (Universal Imaging Corporation, Chester, Pennsylvania).

### Animals

Experiments were performed on male Swiss mice (32 ± 3 g, produced and provided by our local animal facilities, France) in accordance with European communities Council (Directives of November 24, 1986 (86/609/EEC)) and French Legislation (act no. 87-848, Ministère de l'Agriculture et de la Forêt) on Animal Experimentation and were approved by the local ethical committee. Mice were deeply anesthetized with isoflurane 5% and maintained under anesthesia with 2% isoflurane in a 70%/30% gas mixture (N_2_O/O_2_) during surgery. The rectal temperature was maintained at 37 ± 0.5°C throughout the surgical procedure using a feedback-regulated heating system.

### In vivo excitotoxic lesions

A cortical unilateral injection (coordinates: 0.5 mm posterior, 3.0 mm lateral, -0.8 mm ventral to the bregma; stereotaxic atlas G. Paxinos & K.B.J. Franklin) of NMDA (2.5 nmoles; in 0.33 μl), co-injected or not with UBP145 (0.05 nmoles) was performed after placing the animals in a stereotaxic frame. Solutions were injected by the use of a micropipette made with hematologic micropipettes (calibrated at 15 mm/μl; assistant ref 555/5; Hecht, Sondheim-Rhoen, Germany). The micropipette was removed 3 minutes later. After the excitotoxic lesion, a 200 μl bolus intravenous injection of rtPA (10 mg/kg) or saline was performed through a catheter previously inserted into the tail vein.

In a second set of experiments, UBP145 was tested for its ability to cross the blood-brain barrier. In this case, cortical excitotoxic lesions were performed with NMDA, as above, followed by tail vein injections of rtPA alone (10 mg/kg) or in association with UBP145 (pH 7.4; 6.67 mg/kg).

After 24 hours, mice were deeply anesthetized and then transcardially perfused with cold heparinized saline (15 ml) followed by 150 ml of fixative (phosphate buffer 0.1 M. pH 7.4 containing 2% paraformaldehyde and 0.2% picric acid). Brains were post-fixed (18 hours; 4°C) and cryoprotected (sucrose 20% in phosphate buffer; 24 hours; 4°C) before freezing in Tissue-Tek (Miles Scientific, Naperville, IL, USA). Cryomicrotome-cut transversal sections (20 μm) were collected on poly-lysine slides and stained with Fluoro-Jade C [43]. Subsequently, images were captured with a Leica DM6000 microscope-coupled to a coolsnap camera. Cortical lesion surfaces were determined using sections covering 500 μm anterior and posterior to the injection site by using an image analysis system (MetaVue software, Molecular Devices, Downingtown, PA, USA).

### Thrombotic model of stroke and thrombolysis

The same male Swiss mice were placed in a stereotaxic frame and maintained under anesthesia. After a skin incision between the right eye and ear, the master muscle was excised and a small craniotomy (Ø 1 mm) was performed on the parietal bone in order to expose the right middle cerebral artery (MCA). Then, 1 μl of murine α-thrombine (1UI. Sigma Aldrich) was injected in the MCA using hematological micropipettes to activate the coagulation cascade as previously described [30]. To induce thrombolysis, rtPA (10 mg/kg) was injected (200 μl, i.v., 10% bolus and 90% infusion over 40 minutes), 4 hours after MCA occlusion, with or without UBP145 (6.67 mg/kg). Finally, control groups received the same volume of saline with or without UBP145. Cerebral blood velocity was measured by laser-Doppler flowmetry using an optic fiber probe (Oxford Optronix, UK) placed on the skull in the MCA territory. After 24 hours, brains were collected and frozen in isopentane. Then, coronal brain sections (20 μm) were stained with thionine and analyzed with an image analysis software (Image J).

### Statistical analyses

The results are expressed as the mean ± S.E.M. Statistical analyses were performed by the Kruskall-Wallis' test, followed by *post hoc *comparisons, with the Mann-Whitney's test. For calcium videomicroscopy, statistical analyses were performed using Student test. Statistical significances were concluded for p < 0.05.

## List of abbreviations

DIV: days in vitro; DMEM: Dulbecco's modified Eagle's medium; HBBSS: HEPES-buffered saline solution; LDH: lactate dehydrogenase; MAP2: microtubule-associated protein 2; MCA: middle cerebral artery; NMDA: N-methyl-D-aspartate; NMDAR: N-methyl-D-aspartate receptors; PPDA: phenanthrene derivative (2S*, 3R*)-1-(phenanthrene-2-carbonyl)piperazine-2,3-dicarboxylic acid; tPA: tissue plasminogen activator; UBP145: (2R*,3S*)-1-(9-bromophenan-threne-3-carbonyl)piperazine-2,3-dicarboxylic acid

## Competing interests

The authors declare that they have no competing interests.

## Authors' contributions

DTM, EM, DV and CA designed experiments. AJ, AM, CO, FL, DEJ, DV and CA performed and analysed experiments. AJ, AM, FL, DTM, EM, DV and CA wrote and edited the manuscript. All authors read and approved the final manuscript.
